# Quantification of the Impact of Slice Thickness on Volumetric Analysis of Brain Tumors

**DOI:** 10.1002/cam4.72445

**Published:** 2026-10-29

**Authors:** Daniel Vogl, Christer Ruff, Patrick Haas, Georg Gohla, Hanna Gött, Hannes Becker, Marcos Tatagiba, Till‐Karsten Hauser, Constantin Roder

**Affiliations:** ^1^ Department of Neurosurgery University Hospital Tübingen, Eberhard Karls University Tübingen Tübingen Germany; ^2^ Department of Diagnostic and Interventional Neuroradiology University Hospital Tübingen, Eberhard Karls University Tübingen Tübingen Germany; ^3^ Department of Radiology, University Hospital Tübingen Eberhard Karls University Tübingen Tübingen Germany; ^4^ Department of Neurology & Interdisciplinary Neuro‐Oncology University Hospital Tübingen, Hertie Institute for Clinical Brain Research, Eberhard Karls University Tübingen Tübingen Germany; ^5^ Center for Neuro‐Oncology, Comprehensive Cancer Center Tübingen‐Stuttgart University Hospital Tübingen Tübingen Germany

**Keywords:** brain tumor, glioblastoma, magnetic resonance imaging, slice thickness, volumetry

## Abstract

**Background:**

The partial volume effect influences the results of volumetrics performed on MRI sequences with different slice thicknesses. Although a standardized brain tumor imaging protocol with high‐quality 1 mm slices is recommended, segmentation is still commonly performed on thicker slices, especially in retrospective studies. We aimed to quantify the impact of different slice thicknesses on volumetric measurements of brain tumors, using treatment‐naive glioblastoma as a representative example.

**Methods:**

Volumes were measured on T1‐weighted contrast‐enhanced (CE) sequences using semiautomatic segmentation across three predefined slice thicknesses: 1 mm (“thin”; reference measurement), 3 mm (“intermediate”), and 5 mm (“thick”). Data from 46 patients with the availability of all three slice thicknesses were statistically analyzed, including repeated‐measures ANOVA with post hoc comparisons, Bland–Altman analysis, correlations, and linear regression.

**Results:**

The tumor volumes measured increased with slice thickness, with mean values of 36.4 cm^3^ (“thin”), 38.6 cm^3^ (“intermediate”), and 41.0 cm^3^ (“thick”). Repeated measures ANOVA showed significant differences between groups (*p* < 0.01). The mean deviation of the tumor volumes on “thick” relative to “thin” slices was 16% (±10%). Bland–Altman analysis and linear regression confirmed systematically higher measured volumes on “thick” slices. Tumor size on “thin” slices correlated negatively with the percentage deviation on “thick” slices (*r* = −0.56; *p* < 0.01).

**Conclusion:**

Volumetric measurements obtained from “thick” slices showed systematically higher tumor volumes and greater variability relative to the 1 mm reference. Volumetric data obtained from different slice thicknesses should not be combined, or concepts for adjustment should be considered to maintain methodological consistency and reliability in volumetric studies. We advocate the use of high‐quality 3D 1 mm imaging for volumetric analysis whenever possible.

**Abbreviations**
CE: contrast‐enhanced
ICC: Intraclass correlation coefficient
PACS: Picture Archiving and Communication System
RANO: Response Assessment in Neuro‐Oncology group
T: Tesla

## 1 Introduction

Glioblastomas are highly malignant tumors with a poor prognosis despite multimodal treatment concepts [1, 2]. Whenever possible, the first‐line treatment should be surgical resection with maximum safe resection followed by subsequent adjuvant therapy [3]. Despite the availability of multimodal treatment options, glioblastoma remains incurable, and the median postoperative survival time of treated patients in current clinical trials is around 17–24 months [4].

Measurements of tumors have been widely used in medical research, for example, for longitudinal evaluation of growth behavior, calculation of pre‐ and postoperative tumor volumes, or to assess the influence of radiation/chemotherapy on tumor recurrence [5, 6]. Particularly in neuro‐oncology, the measurement of brain tumor size plays an important role in preoperative treatment planning, evaluation of the extent of resection after tumor resection, or response assessment [7, 8, 9]. Various approaches for measuring tumor size have been proposed, primarily in the context of developing and defining response criteria [10, 11]. Historically, tumors have been measured on MRI using linear methods due to their simplicity and unavailability of digital data and measurement tools. Measurement using the largest diameter can be unidimensional or bidimensional, and measurement by calculating an ellipsoid using tumor diameters in three dimensions has also been frequently used [10, 11, 12]. Nowadays, volumetric, 3‐dimensional digital measurements can be easily obtained using image processing software packages with semiautomatic segmentation algorithms [13]. A recently published update from the Response Assessment in Neuro‐Oncology group (RANO) recommends two‐dimensional (the maximum cross‐sectional area of tumor) or volumetric measurement as an alternative for assessing gliomas [8].

Volumetry using segmentation software, such as the commercially available Brainlab SmartBrush (Brainlab AG, Munich, Germany) or open‐source software such as 3D Slicer [14], is based on marking voxel intensities, irrespective of manual, semiautomatic, automatic, or AI‐supported workflows [13]. Including all affected voxels in the segmentation has the advantage of capturing irregular tumor shapes, as glioblastomas most often do not show sharply demarcated round or ellipsoid growth shapes. However, this 3‐dimensional method is likely to distort tumor volume measurements at greater slice thicknesses due to the partial volume effect [15]. This effect becomes apparent when more than one tissue type occurs in a voxel. The partial volume effect is well understood in the context of MR principles and has been extensively researched in the past. Although this is relevant to everyday clinical practice, the effect of slice thickness on brain tumor volumetry has not yet been quantified in neurosurgical research [15, 16].

While tumor visualization on MR imaging used to be performed mainly on two‐dimensional (2D) scans with slice thicknesses of up to 6 mm and, due to cross‐talk, with a distance factor, three‐dimensional (3D) high‐quality imaging with 1 mm slice thickness without spacing between slices is now state‐of‐the‐art in perioperative neuroradiologic diagnostics [3, 17, 18]. However, especially for MR diagnostics performed outside the high‐volume centers, routine acquisition of 3D sequences has not yet become the standard practice, potentially due to time and cost factors [19]. Although there is some knowledge about the inaccuracy of measurements with thickly sliced sequences, MR sequences with a slice thickness of up to 5 mm are still commonly used in clinical studies, mainly in retrospective analyses. This study aimed to quantify the magnitude and variability of differences in semiautomatically segmented tumor volumes across clinically acquired MRI datasets with different slice thicknesses, using untreated glioblastoma cases as a representative example.

## 2 Materials and Methods

### 2.1 Study Design and Patients

This study is a retrospective analysis to evaluate the impact of different MRI slice thicknesses on volumetric measurement results of brain tumors. To quantify these potential differences, three categories of slice thickness were defined: “thin” (1 mm; reference measurement), “intermediate” (3 mm), and “thick” (4–5 mm). To ensure methodological consistency, only patients with newly diagnosed, treatment‐naïve glioblastoma were enrolled.

Medical records of patients with untreated, histologically proven glioblastoma (IDH‐wildtype, WHO grade 4; as per WHO 2021 classification) from 2015 to 2020 (366 patients) were screened for availability of MRI data with different slice thicknesses of contrast‐enhanced (CE) T1‐weighted sequences (T1CE) stored in the hospital's Picture Archiving and Communication System (PACS). Demographic and clinical data were acquired from patients' medical records. The study protocol was approved by the local ethics committee, with a waiver of consent (as the study was retrospective and used previously acquired data collected as part of routine care). The data collection was done in line with the Declaration of Helsinki.

### 2.2 MR Imaging

To eliminate potential differences in image acquisitions between different manufacturers, we only chose MRI data acquired on Siemens Healthineers (Erlangen, Germany) scanners with 1.5 or 3T; 3T scanners included Magnetom Prisma and Magnetom Skyra, and 1.5T scanners included the Magnetom Espree, Magnetom Avanto, Magnetom Aera, and Magnetom Symphony models. MR protocols had to comprise a 3‐dimensional, isotropic magnetization‐prepared rapid acquisition gradient echo (MPRAGE) T1‐weighted sequence, a multiplanar reconstruction (3 mm slice thickness), and a 2D T1‐weighted (turbo) spin‐echo sequence (4–5 mm slice thickness) after administration of a gadolinium contrast medium [17].

Volumetric analyses for each of the aforementioned slice thicknesses were conducted on T1CE by the first author (D.V.) and reviewed by the senior author (C.R.) using the segmentation tool of Brainlab Elements SmartBrush (version 4.5.0.71; Brainlab AG, Munich, Germany). The semiautomatic segmentation was manually verified on each slice and corrected if necessary.

### 2.3 Statistical Analysis

All statistical analyses were conducted using IBM SPSS Statistics version 28.0 (IBM Corp., Armonk, NY, USA). For statistical tests, a *p*‐value < 0.05 was considered statistically significant.

Mean values (±SD) and medians of absolute and relative tumor volumes for each slice‐thickness group were analyzed descriptively. Percentage volumetric deviations were calculated using “thin” as the reference variable. Differences in mean values were tested for statistical significance using repeated measures ANOVA. The normal distribution of data was assessed using the Shapiro–Wilk test. A square root transformation was used for right‐skewed data. To evaluate differences between pairs of slice thicknesses, paired *t*‐tests were conducted. The intraclass correlation coefficient (ICC) with a two‐way mixed model for absolute agreement was calculated to assess the consistency of volumetric measurements across different slice thicknesses [20].

Bland–Altman plots were generated to assess the agreement between volumetric measurements obtained on different slice thicknesses. The mean differences and limits of agreement (±1.96 SD) were calculated for each pairwise comparison (“thin” vs. “intermediate”, “thin” vs. “thick”, and “intermediate” vs. “thick”). The analysis focused on identifying systematic bias or variability introduced by different slice thicknesses.

Connected dot plots were used to visualize the differences between volumetric results of slice thicknesses in terms of the absolute volumes of “thin” (serving as the reference) and the relative deviation of volumetric measures performed on corresponding “intermediate” and “thick” slices. Scatter plots of the absolute tumor volume of “thin” and the relative tumor volume deviation of larger slice thicknesses were depicted. Their relationship was assessed using Pearson's correlation (*r*). Linear regression models were created for pairwise comparisons (“thin” vs. “intermediate”; “thin” vs. “thick”).

## 3 Results

The general characteristics of the 46 patients included are presented in Table 1. The mean patient age was 63 years, and sex was equally distributed. Twenty‐seven tumors (59%) were located on the right side, 16 (35%) on the left side, and 3 (6%) extended to both hemispheres. The majority of tumors were located in the frontal (37%), temporal (26%), or parietal lobe (24%). The field strength of the MR scanners was 1.5T in 31 patients (67%) and 3T in 15 patients (33%).

**TABLE 1:** Patient characteristics.

Parameter	Patients (*n* = 46)
Age (y)	63 ± 3
Sex	
Male (%)	23 (50)
Tumor side (%)	
Right	27 (59)
Left	16 (35)
Midline	3 (6)
Tumor localization (%)	
Frontal	17 (37)
Temporal	12 (26)
Parietal	11 (24)
Occipital	1 (2)
Butterfly glioma	2 (4)
Insular	2 (4)
Pineal	1 (2)
Field strength	
1.5T (%)	31 (67)
Magnetom Espree	3
Magnetom Avanto	13
Magnetom Aera	11
Magnetom Symphony	4
3T (%)	15 (33)
Magnetom Prisma	7
Magnetom Skyra	8

### 3.1 Impact of Slice Thickness on Volumetric Measurements

The mean preoperative tumor volumes for “thin”, “intermediate,” and “thick” slices were 36.4 ± 27.8 cm^3^, 38.6 ± 29.1 cm^3^, and 41.0 ± 30.4 cm^3^, respectively (*p* < 0.001, Table 2). Median values were 29.3 cm^3^, 31.1 cm^3^, and 32.3 cm^3^. “Intermediate” slice thickness exhibited a mean relative deviation of 7.1% (±4.0%), with an average absolute difference of 2.1 cm^3^ (±1.7 cm^3^) relative to the reference (1 mm sequences). In comparison, “thick” slices showed an average absolute difference of 4.5 cm^3^ (±3.7 cm^3^) and a mean relative deviation of 16.0% (±10.0%) compared to “thin” slices. A subgroup analysis by field strength showed a higher mean relative deviation particularly for “thick” slices acquired on 1.5T scanners compared with 3T scanners (19% ± 10% vs. 10% ± 5%; *p* < 0.001; Table S1).

**TABLE 2:** Analysis of volumetric results across different slice thickness.

Parameter	Slice thickness			*p*	Bland–Altman analysis		Within‐participants coefficient of variation CV %	Intraclass correlation coefficient (ICC)	
	Thin	Intermediate	Thick		Difference	LOA		Single (95% CI)	Average (95% CI)
Mean (cm^3^)	36.4 ± 27.8	38.6 ± 29.1	41.0 ± 30.4	< 0.001^a^	−2.1 (Thin, Int.)	−5.5 – 1.3	4.7	0.995 (0.992–0.997)	0.998 (0.997–0.999)
Median (cm^3^; min.; max.)	29.3 (3.2;116)	31.1 (3.5;121)	32.3 (4.0;130)		−4.5 (Thin, Thick)	−11.9 – 2.8	9.7		
Mean absolute deviation (cm^3^)	reference	2.1 ± 1.7	4.5 ± 3.7		−2.4 (Int., Thick)	−7.5 – 2.8	6.6		
Mean relative deviation (cm^3^; %)		7.1 ± 4	16 ± 10						

Abbreviations: CI = Confidence Interval, CV = Coefficient of variation, ICC = Intraclass correlation coefficient, LOA = Limits of Agreement.

^a^ Repeat measures ANOVA.

### 3.2 Agreement in Tumor Volume Measurements

Despite significant differences in absolute tumor volumes, the ICC showed good agreement between the three groups (0.995; Table 2). These significant differences were further analyzed pairwise, showing highly significant differences in all tests (paired *t*‐test, *p* < 0.001 in all three comparisons; Figure S1).

Figure 1 illustrates how volumetric measurements vary with slice thickness and highlights the overestimation of tumor volumes associated with segmentation performed on thicker slices. While tumor volumes of “intermediate” slices tend to rather align with volumes acquired on “thin” slices, volumes of “thick” slices show higher values and greater variability. The mean within‐participant coefficient of variation was 4.8% (0.7%–11.7%) for “thin” vs. “intermediate”, 10.2% (1.2%–23.9%) for “thin” vs. “thick” and 5.4% (0%–15.6%) for “intermediate” vs. “thick” (Figure 1A, Table 2). Figure 1B shows relative deviations with “thin” as the reference category, highlighting the variability of volumes obtained from “thick” slices.

**FIGURE 1:**
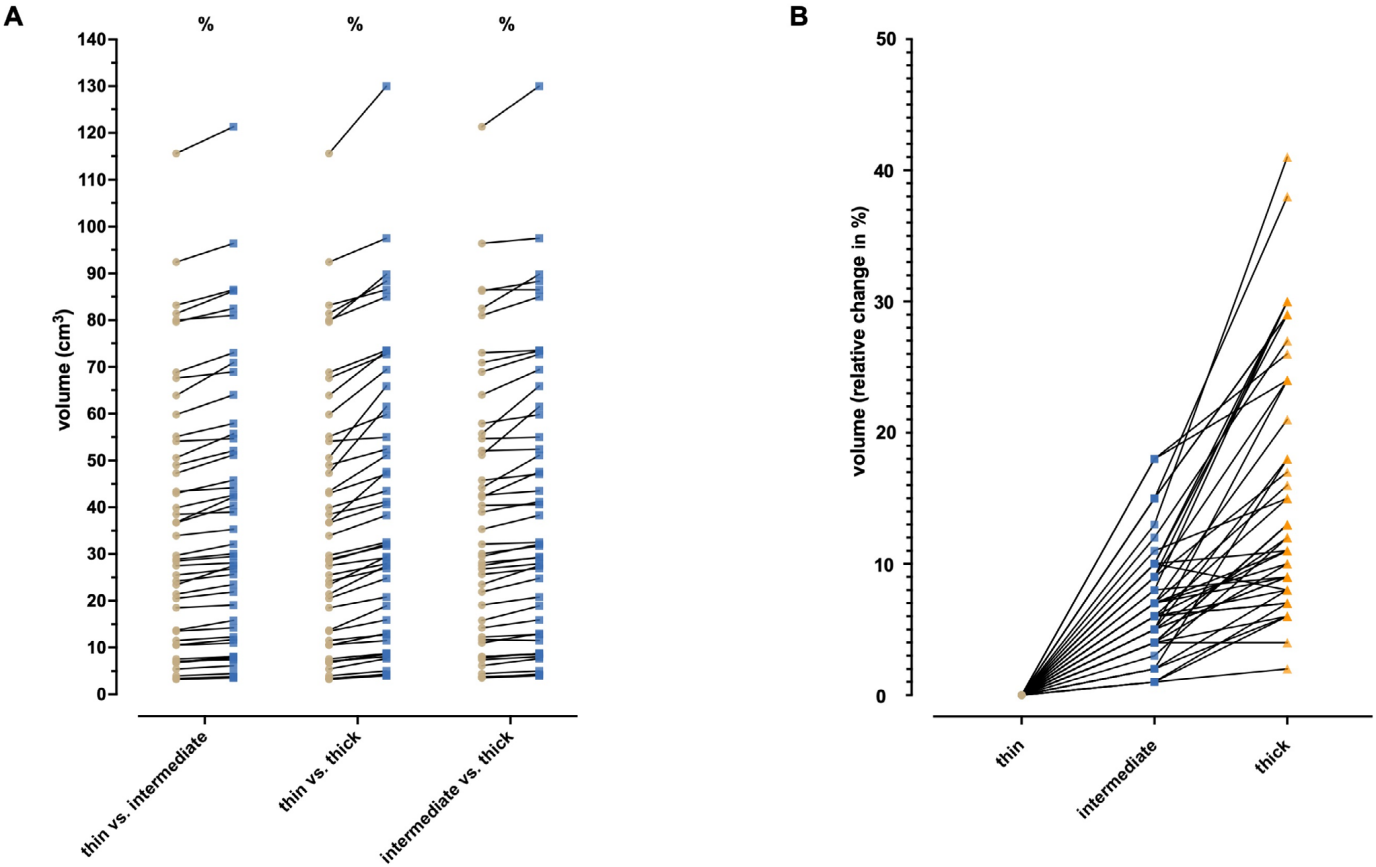
Paired line plots comparing volumetric measurements across “thin”, “intermediate,” and “thick” slices. The left panel reveals pairwise comparisons (“thin” vs. “intermediate,” “thin” vs. “thick,” and “intermediate” vs. “thick”), highlighting systematic differences. The mean within‐participant coefficient of variation (CV) was 4.8% for “thin” vs. “intermediate,” 10.2% for “thin” vs. “thick,” and 5.4% for “intermediate” vs. “thick”. The right panel shows relative deviations with “thin” as the reference category.

The Bland–Altman analysis (Figure 2, Table 2) revealed the constant overestimation of tumor volume with increasing slice thickness. The plots show differences against mean values, with mean biases of −2.1 cm^3^ (limits of agreement: −5.5 cm^3^; 1.3 cm^3^), −4.5 cm^3^ (−11.9 cm^3^; 2.8 cm^3^), and −2.4 cm^3^ (−7.5 cm^3^; 2.8 cm^3^) for the slice thicknesses “thin” vs. “intermediate”, “thin” vs. “thick”, and “intermediate” vs. “thick”, respectively. The 95% limits of agreement indicate that “thick” slices in particular show a positive bias in measurements.

**FIGURE 2:**
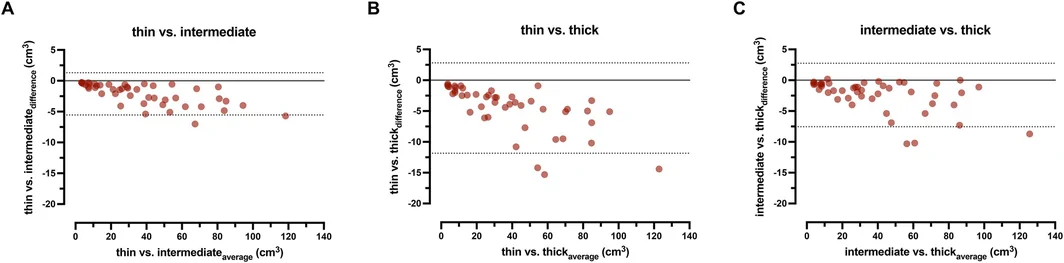
Bland–Altman plots comparing absolute volumes of different measurement methods: “thin” vs. “intermediate” (left), “thin” vs. “thick” (center), and “intermediate” vs. “thick” (right). The *y*‐axis shows the differences between groups of slice thickness, plotted against their mean values (*x*‐axis). Dashed lines: 95% limits of agreement (±1.96 SD); solid line: Mean difference.

### 3.3 Correlation and Regression Analysis

A strong positive correlation was found between all three groups (“thin” – “intermediate”: *r* = 0.999, “thin” – “thick”: *r* = 0.995, and “intermediate” – “thick”: *r* = 0.997; Figure S2). Linear regression analysis quantified these relationships. The predicted overestimation of 8.2% for “thick” slices (derived from the regression coefficient of the equation) aligns well with the results presented above.

The values of the relative tumor volume deviation of “intermediate” and “thick” sequences correlated negatively with absolute volumes of “thin” sequences (abs. “thin” – rel. “intermediate”: *r* = −0.46, *p* < 0.01; abs. “thin” – rel. “thick”: *r* = −0.56, *p* < 0.01; Figure 3). These findings indicate a tendency for volumetric measurements based on thicker imaging slices to overestimate tumor size, particularly in smaller lesions.

**FIGURE 3:**
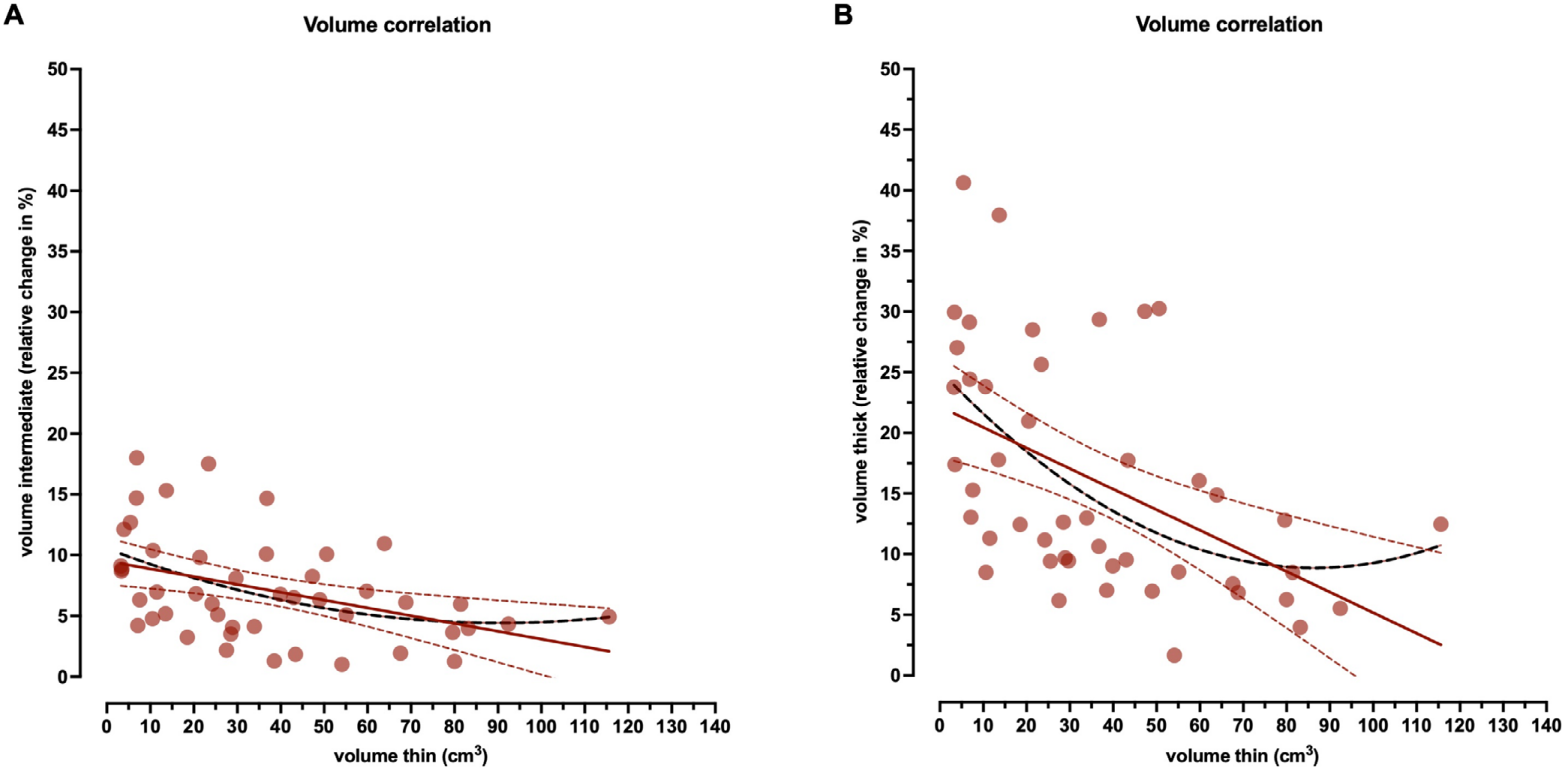
Scatter plots showed statistically significant negative correlations between absolute tumor volumes measured on “thin” slices (*x*‐axis) and relative volume deviations on “intermediate” (A) and “thick” (B) slices (*y*‐axis): (A) abs. volume “thin” – rel. volume “intermediate”: *R* = −0.46, *p* < 0.001 and (B) abs. volume “thin” – rel. volume “thick”: *R* = −0.56, *p* < 0.001.

## 4 Discussion

Volumetric segmentation of brain tumors on MRI sequences is frequently employed in research and in clinical practice for various reasons. An internationally recognized protocol for standardized datasets in clinical trials recommends the acquisition of 3D sequences for brain tumors [17]. However, the corresponding scan must be planned for a 30‐min time slot, and both clinical experience and findings from a large European survey indicate that some patients with intracranial tumors still undergo low‐quality imaging, often based on protocols using greater slice sequences rather than high‐resolution 3D datasets [19]. As there is a trade‐off between scan time and high‐resolution imaging in conventional MR imaging, the reasons for this may be influenced by economic factors, such as time pressure and financial considerations [19].

This study aimed to quantify the effect of varying slice thickness (1 mm = “thin”, 3 mm = “intermediate,” 4–5 mm = “thick”) on volumetric measurements. To ensure methodological homogeneity, the study was confined to patients with treatment‐naïve glioblastoma; however, we believe the conclusions may also apply to other tumor entities and post‐therapeutic imaging.

We found that tumor volumes increased with increasing slice thickness, with mean volumes of 36.4 cm^3^, 38.6 cm^3^, and 41.0 cm^3^ for “thin”, “intermediate,” and “thick” slices, respectively. Mean differences relative to “thin” slices were 2.1 cm^3^ and 4.5 cm^3^ for “intermediate” and “thick” slices, respectively. These differences might mainly be attributed to the partial volume effect. This effect occurs when different tissue types within a voxel are averaged, leading to variations in measurement volumes at different slice thicknesses [15]. Particularly, “thick” slices (4–5 mm) showed high variability in the measurements. An average deviation from the reference measurement (1 mm) of approximately 16% was observed, with a maximum overestimation of 41% and a standard deviation of 10%. These findings align with previous calculations by Ballester et al. [15, 16], who estimated inaccuracies ranging from 20% to 60% when ignoring the partial volume effect. A recent study that resampled 3D CE T1 datasets to larger voxel sizes (similar to our intermediate group) reported a similar pattern. As voxel size increased, volumes became progressively larger. At the thickest voxel size (1.2 × 0.9 × 4.0 mm), the average volume deviation was about 30% [21]. Importantly, the association of slice thickness with volumetric accuracy has also been demonstrated in experimental phantom studies using MRI acquisitions of objects with independently determined true volumes [22, 23, 24]. These studies demonstrated increasing volumetric measurement errors with greater slice thickness, providing experimental support for the slice‐thickness dependency observed in our clinical cohort.

Variations in tumor measurements across different slice thicknesses are likely to occur primarily at the tumor margins. On MR imaging, glioblastomas typically present with a CE rim surrounding a central area of necrosis. This rim is mostly irregularly shaped and can only be delineated precisely by high‐resolution, thin‐slice imaging techniques such as 3D MPRAGE, which facilitates the definition of accurate tumor boundaries. In contrast, sequences utilizing thicker slice thickness lose inter‐slice information by averaging tissue signal, particularly in the rim regions, leading to significantly worse delineations of tumor borders [21]. Substantial variability in volumes acquired on thicker slices than thinner slices was observed. This variation may be attributed to the previously mentioned irregularly shaped tumors, where the loss of structural detail in between slices is more pronounced than in more homogeneous, spherical tumors [22]. Furthermore, as exclusively larger volumes in “intermediate” and “thick” slices compared to “thin” slices were observed, we concluded that interpolation at the tumor margin consistently results in overestimation rather than underestimation of tumor volume.

Although the absolute deviation was greater in larger tumors, the percentage deviation was greater in smaller tumors. In this regard, we found a moderate negative correlation between tumor volumes measured on “thin” slices and the percentage deviation observed on “thick” slices (*r* = −0.56, *p* < 0.01). Consequently, in particular smaller tumors tend to be overestimated by volumetry performed on thicker slices, which might be caused by a higher surface area to volume ratio [21, 25]. Despite significant differences in absolute volumes, the ICC showed good agreement (0.995). This may seem to conflict with the finding of significant differences in absolute tumor volumes at different slice thicknesses. However, it also indicates that volumetry performed on “intermediate” and “thick” slices systematically leads to higher volumetric values for all tumors. In other words, this emphasizes the importance of maintaining consistency in slice thickness for longitudinal, intra‐individual volumetric tumor measurements. Therefore, comparing segmentations performed on 3D MR sequences with thicker (2D) sequences should be avoided. For example, in the context of response assessment, an apparent increase in residual tumor size could be attributed to thicker slices in follow‐up imaging (e.g., influenced by cost‐effectiveness considerations at out‐of‐hospital facilities) compared to the high‐quality postoperative baseline MRI, rather than reflecting actual tumor growth.

Although the described inaccuracy associated with thick slices in volumetric measurements is well known, few studies actually implement corrective measures to account for this, for example by calculating limits of agreement for longitudinal tumor volumetrics at different slice thicknesses [26]. Methods to correct for data obtained with varying slice thicknesses to improve comparability (e.g., based on mathematical models) may be considered, particularly in studies in which different imaging protocols are likely, such as retrospective data with various MR protocols or even multi‐center trials [27]. If thicker datasets must be used for volumetric assessment, AI‐based segmentation may provide more accurate results than manual segmentation [21].

The current trend in glioblastoma research is moving away from the sole assessment of the CE tumor and toward the evaluation of the non‐CE component, measured on the fluid‐attenuated inversion recovery (FLAIR) sequence or PET MRI [28, 29, 30]. Furthermore, volumetric 3D assessment is gaining increasing importance in grade 1–3 glioma clinical trials [31]. The variability in volumetric measurements on different slice thicknesses caused by the partial volume effect, observed in this study on T1‐weighted CE images, could also apply to T2‐weighted sequences, including FLAIR. This is expected considering the underlying MR physics, as the mechanism is primarily linked to voxel geometry and should therefore apply across different pulse sequences. In addition, a study comparing 2D versus isotropic 3D FLAIR has reported significant differences in measured lesion volumes [32]. However, standardized brain tumor imaging protocols recommend 2D FLAIR sequences with a slice thickness of 4 mm, while simultaneously 3D FLAIR sequences are increasingly used in clinical routine, owing to their higher spatial resolution and potential use for neuronavigation [17, 33, 34]. Given the growing significance of volumetric analysis of T2/FLAIR imaging for the non‐CE tumor compartment of glioblastoma, as well as in low‐grade tumors, our results indicate that further standardization of both imaging protocols and volumetric measurement (also concerning the use of artificial intelligence) is necessary for obtaining reproducible and reliable volumetric results in future retrospective and prospective trials that include tumor volumetry [8, 35].

Considering the aforementioned economic considerations, the acquisition of a 3D dataset with 1 mm slice thickness primarily increases operational costs due to a longer scanner occupancy time with potential effects on throughput. From a sustainability perspective, longer scan times increase energy use [36]. In the context of current discussions and research on sustainable radiology, shortening examination times is a key strategy for reducing energy consumption [37]. Contemporary acceleration strategies (parallel imaging/compressed sensing, and deep‐learning‐based reconstruction) can substantially shorten 2D and high‐resolution 3D acquisitions, potentially improving feasibility in routine time slots while simultaneously improving image quality [38, 39].

The study findings should be interpreted cautiously in light of several limitations. A primary limitation is the retrospective nature of this study. This design allowed us to assess volumetric differences in imaging datasets representative of routine clinical practice and retrospective research. However, it limited our ability to standardize acquisition parameters, such as in‐plane resolution, and isolate their individual effects. Previous research on the effect of in‐plane resolution on volume measurements has shown that small changes in spatial resolution do not affect these measurements [40]. Andica et al. demonstrated that for the majority of brain tissues, there were no significant differences in the measured volumes between different in‐plane resolutions (0.8 vs. 2.0 vs. 3.0 mm). Saccenti et al. also revealed no significant differences in measured volumes between the 0.8 and 1.8 mm in‐plane resolutions [41]. Therefore, it might be assumed that different in‐plane resolutions used in this study most likely did not significantly impact tumor volumetry. However, a certain degree of formal uncertainty remains. This also applies to potential image quality variations caused by the different time of acquisition between 2D and 3D sequences and time delay of contrast enhancement [42]. In the acquisition protocol, the 2D CE T1‐weighted sequence with thicker slices was obtained first, followed by the 3D 1 mm CE T1‐weighted sequence. Consequently, any time‐dependent increase in CE due to continued contrast leakage/diffusion would be expected to increase volumes on the later 3D sequences, not on the earlier 2D acquisition. Nevertheless, to control these time‐dependent effects, a methodological alternative would be to create thick slices by reconstructing the 1 mm 3D CE dataset, analogous to the 3 mm reconstruction (intermediate group) of this study. Results of a recent study using this approach were consistent with our findings [21]. However, neither such resampling approaches nor our study can provide an independent ground‐truth tumor volume. The 1 mm sequences therefore serve as a reference measurement rather than a measure of the true biological tumor volume. Consequently, the observed differences indicate a relative measurement deviation between imaging datasets but do not determine the absolute accuracy of either acquisition. In this regard, previous phantom studies using objects with independently known volumes have demonstrated that volumetric accuracy decreases with increasing slice thickness [22, 23, 43]. As our goal was to reflect routine clinical workflows, we ultimately chose to evaluate separately acquired thin‐ and thick‐slice sequences. As only one software package was used for tumor volumetry, the application of another volumetric analysis tool could yield different results [44].

In conclusion, this study demonstrated systematically higher measured tumor volumes with a mean deviation of 16% on “thick” slices (4–5 mm slice thickness) relative to “thin,” 1 mm reference acquisitions. Thick slices additionally showed notable variability. These findings support the use of high‐quality 1 mm, 3D imaging for volumetric assessment whenever possible and emphasize the importance of consistent acquisition protocols. Volumetric measurements obtained from datasets with different slice thicknesses should not be considered interchangeable without accounting for potential measurement differences.

## Author Contributions

**Daniel Vogl:** conceptualization, methodology, data curation, investigation, formal analysis, visualization, writing – original draft, writing – review and editing, project administration. **Christer Ruff:** conceptualization, methodology, validation, formal analysis, visualization, writing – review and editing, resources, investigation. **Patrick Haas:** formal analysis, writing – review and editing, methodology. **Georg Gohla:** writing – review and editing, methodology, validation, resources. **Hanna Gött:** writing – review and editing. **Hannes Becker:** writing – review and editing. **Marcos Tatagiba:** supervision, writing – review and editing, resources. **Till‐Karsten Hauser:** conceptualization, methodology, validation, resources, writing – review and editing. **Constantin Roder:** conceptualization, methodology, data curation, supervision, validation, project administration, resources, writing – review and editing, investigation.

## Funding

This work was not supported by any grant. We acknowledge support from the Open Access Publication Fund of the University of Tübingen.

## Conflicts of Interest

The authors declare no conflicts of interest.

## Supporting information


**Table S1:** Group differences between field strength of MRI scanners.
**Figure S1:** Scatter plots of absolute tumor voluminal across different slice thicknesses. Paired t‐test comparing ST: “Thin” vs. “intermediate”: *p* < 0.001; “Thin” vs. “large”: < 0.001; “Intermediate” vs. “large”: *p* < 0.001.
**Figure S2:** Scatter plots for absolute (abs.) tumor volumes across the three groups of different slice thickness. A strong positive correlation can be observed: “Thin” – “Intermediate”: *r* = 0.999, “Thin” – “Thick”: *r* = 0.995, and “Intermediate” – “Thick”: *r* = 0.997; *p* < 0.001 for all.

## Data Availability

De‐identified data generated or analyzed during this study are available from the corresponding author upon reasonable request.
