# Supplementary material for: Quantification of the Impact of Slice Thickness on Volumetric Analysis of Brain Tumors
Source: Cancer Med. 2026 Oct 29;15(11):e72445. doi: 10.1002/cam4.72445 (PMC13717382; doi:10.1002/cam4.72445)
Supplement: Supplementary file 1 [file CAM4-15-e72445-s001.docx]

**Supplementary table 1. Group differences between field strength of MRI scanners**

|  | Field Strength | | |  |
| --- | --- | --- | --- | --- |
|  | 1.5 Tesla (n = 31) | 3 Tesla (n = 15) | p value |  |
| Thin (1mm) |  |  |  |  |
| Mean | 32.3 ± 23.5 | 44.8 ± 34.9 | 0.28 ^a^ |  |
|  |  |  |  |  |
| Intermediate (3mm) |  |  |  |  |
| Mean (cm^3^) | 34.7 ± 24.7 | 46.5 ± 36.3 | 0.32 ^a^ |  |
| Mean Relative Deviation (%) | 8 ± 6 | 5 ± 3 | 0.01 ^a^ |  |
|  |  |  |  |  |
| Thick (4mm – 5mm) |  |  |  |  |
| Mean (cm^3^) | 37.2 ± 27.7 | 48.8 ± 38.1 | 0.36 ^a^ |  |
| Mean Relative Deviation (%) | 19 ± 10 | 10 ± 5 | <0.001 ^a^ |  |

^a^ = χ2 - test

Supplementary Figure 1: Scatter plots of absolute tumor voluminal across different slice thicknesses. Paired t-test comparing ST: “Thin” vs. “intermediate”: p<.001; “Thin” vs. “large”: <.001; “Intermediate” vs. “large”: p<.001

Scatter plots of tumor volumes showed that values “Thick” slices tended to be higher than the values for “thin” and “intermediate”. Though visually not clearly distinguishable, significant differences were found in paired t-tests across all groups.

Supplementary Figure 2: Scatter plots for absolute (abs.) tumor volumes across the three groups of different slice thickness. A strong positive correlation can be observed: “Thin” – “Intermediate”: r = 0.999, “Thin” – “Thick”: r = 0.995, and “Intermediate” – “Thick”: r = 0.997; p<0.001 for all

Calculated regression equations:

abs.Volume(“Thick”) =1.082 x abs.Volume(“Thin”) +1.530

abs.Volume(“Intermediate”) =1.042 x abs.Volume(“Thin”) +0.606
